# DNA metabarcoding assays reveal a diverse prey assemblage for *Mobula* rays in the Bohol Sea, Philippines

**DOI:** 10.1002/ece3.4858

**Published:** 2019-01-30

**Authors:** Cindy Bessey, Simon N. Jarman, Michael Stat, Christoph A. Rohner, Michael Bunce, Adam Koziol, Matthew Power, Joshua M. Rambahiniarison, Alessandro Ponzo, Anthony J. Richardson, Oliver Berry

**Affiliations:** ^1^ Indian Oceans Marine Research Centre Commonwealth Scientific and Industrial Research Organization, Oceans and Atmosphere Crawley Western Australia Australia; ^2^ Trace and Environmental DNA Laboratory, School of Molecular and Life Sciences Curtin University Perth Western Australia Australia; ^3^ School of Plant Biology and the Oceans Institute University of Western Australia Crawley Western Australia Australia; ^4^ Environomics Future Science Platform, Indian Oceans Marine Research Centre Commonwealth Scientific and Industrial Research Organization Crawley Western Australia Australia; ^5^ Department of Biological Sciences Macquarie University North Ryde New South Wales Australia; ^6^ Marine Megafauna Foundation Truckee California; ^7^ Large Marine Vertebrates Research Institute Philippines Bohol Philippines; ^8^ Commonwealth Scientific and Industrial Research Organization, Oceans and Atmospheres Brisbane Queensland Australia; ^9^ Centre for Applications in Natural Resource Mathematics, School of Mathematics and Physics University of Queensland St Lucia Queensland Australia

**Keywords:** manta rays, metabarcoding, Myliobatidae, prey

## Abstract

Diet studies provide base understanding of trophic structure and are a valuable initial step for many fields of marine ecology, including conservation and fisheries biology. Considerable complexity in marine trophic structure can exist due to the presence of highly mobile species with long life spans. *Mobula *rays are highly mobile, large, planktivorous elasmobranchs that are frequently caught either directly or as bycatch in fisheries, which, combined with their conservative life history strategy, makes their populations susceptible to decline in intensely fished regions. Effective management of these iconic and vulnerable species requires an understanding of the diets that sustain them, which can be difficult to determine using conventional sampling methods. We use three DNA metabarcode assays to identify 44 distinct taxa from the stomachs (*n* = 101) of four sympatric *Mobula* ray species (*Mobula birostris*, *Mobula tarapacana*, *Mobula japanica*, and *Mobula thurstoni*) caught over 3 years (2013–2015) in a direct fishery off Bohol in the Philippines. The diversity and incidence of bony fishes observed in ray diets were unprecedented. Nevertheless, rays showed dietary overlap, with krill (*Euphausia*) dominating their diet. Our results provide a more detailed assessment of sympatric ray diets than was previously described and reveal the complexity that can exist in food webs at critical foraging habitats.

## 1 INTRODUCTION

Diet studies provide basic knowledge of a species’ diet composition, its trophic position, and the links between predator and prey in the food web. But dietary information has use beyond pure ecology in a variety of applied studies. Trophic connectivity informs ecosystem‐based fisheries models, which aim to sustain a healthy marine ecosystem and thus support fisheries (Hollowed et al., 2000; Pikitch et al., 2004), as changes in one part of the food web have wider implications (Estes et al., 2011; Pompanon et al., 2012). Foraging and feeding are also key drivers for movements, and understanding habitat use is important for managing and conserving stocks (Block et al., 2011). Dietary information can be directly applied to help reduce incidental catch in protected species, for example, by changing the type of bait used when fishing (Watson, Epperly, Shah, & Foster, 2005). Multispecies feeding studies examine dietary overlap (Foley, Bowen, Nalepa, Sepulveda, & Hook, 2014; Jackson et al., 2016; Stewart et al., 2017) and trophic niche partitioning (Cherel, Hobson, Guinet, & Vanpe, 2007), which have further implications for the competition of prey among sympatric species. Ecology, conservation biology, and fisheries rely on food web characterization as an initial step in ecosystem understanding.

For marine animals including fish, cephalopods, crustaceans, seabirds, and mammals, the traditional way of identifying the dietary linkages of a species is through gut contents analysis by light microscopy (Richardson, Lamberts, Isaacs, Moloney, & Gibbons, 2000). A major limitation of stomach contents analysis is that prey items are often digested, making them difficult or impossible to identify microscopically. This also introduces a bias toward recognizing organisms with hard parts that are resistant to digestion (Berg, 1979). More recently, molecular approaches are being used to identify the often digested prey of marine animals (Berry et al., 2015). These approaches have the benefit of being able to identify to species heavily digested fragments, providing exciting new insights into the dietary diversity of marine animals.

*Mobula* rays, commonly known as manta and devil rays, are a genus of large, iconic, and highly mobile, planktivorous elasmobranchs from the family Myliobatidae (Bonaparte, 1835), with a global distribution in tropical to warm‐temperate waters (Couturier et al., 2012; Van Der Laan et al., 2014). There is considerable variation in the size of rays within this genus; the giant manta ray *Mobula birostris* can reach a maximum disk width of over 900 cm (Croll et al., 2016), while the bentfin devil ray *Mobula thurstoni* grows to ~200 cm disk width (Couturier et al., 2012). Although their life span is unknown, studies estimate their longevity to be>14 years (*Mobula japanica*; Cuevas‐Zimbrón, Sosa‐Nishizaki, Pérez‐Jiménez, & O’Sullivan, 2012) or longer (40 years for *M. birostris*; Marshall et al., 2011). *Mobula* rays are aplacental viviparous, with an estimated gestation period of 1 year (Marshall & Bennett, 2010; Notarbartolo‐Di‐Sciara, 1988). They typically give birth to a single pup with a possible resting period of 2–5 years between pregnancies (Croll et al., 2016; Marshall & Bennett, 2010) and may delay the age of first reproduction when food is scarce during their development (Couturier et al., 2012). This life history strategy makes them susceptible to overexploitation (Croll et al., 2016; Dulvy, Pardo, Simpfendorfer, & Carlson, 2014).

Fishing pressure, both directly and as bycatch, is a major threat to many *Mobula* populations. They are targeted for their gill plates which are used in traditional medicine, for food and local products in artisanal fisheries, and incidentally captured in gill, purse‐seine and trawl nets, and on long‐lines (Couturier et al., 2012; Croll et al., 2016; Rajapackiam, Mohan, & Rudramurthy, 2007). As a result, many *Mobula* rays are currently listed as “vulnerable” or “near‐threatened” by the IUCN (International Union for Conservation of Nature), and all species have been added to CITES (Convention on the International Trade in Endangered Species) Appendix II https://www.cites.org/eng/app/appendices.php and CMS (Convention on the Conservation of Migratory Species of Wild Animals) Appendices I and II https://www.cms.int/en/page/appendix-i-ii-cms.

Fisheries managers are now adopting ecosystem‐based approaches in resource management, which requires a basic knowledge of trophic interactions. Since predator–prey interactions are difficult to observe directly, dietary studies are a common method used to determine feeding ecology and trophic dynamics (Brodeur, Smith, McBride, Heintz, & Farley, 2017). Resolving the diets of *Mobula* rays, which encompass a wide range of body sizes and converge in specific locations, can help characterize trophic links in critical foraging areas.

Very few studies have described the diet of sympatric *Mobula* rays. Dietary analysis of *Mobula* species has been conducted using microscopy of stomach contents (Notarbartolo‐Di‐Sciara, 1988; Rohner et al., 2017), as well as stable isotope analyses (Sampson, Galvan‐Magana, Silva‐Davila, & Aguiniga‐Garcia, 2010; Stewart et al., 2017). Microscopy provides detailed information on taxa consumed for a particular individual at a specific period of time (Hyslop, 1980), while stable isotope analysis provides insights into relative trophic level and the sources of carbon supporting diets, that are integrated over time (Peterson & Fry, 1987). All previous microscopy studies of *Mobula* species identified Euphausiids (krill) as the dominant (>90%) prey item for four species (*M. birostris*, *Mobula tarapacana*, *M. japanica,* and *M. thurstoni*), over all locations and ray sizes, with stable isotope studies indicating they were second level consumers with large overlap in their isotopic niche space. Few fish species have been identified as prey items, with the exception of *M. birostris* stomachs containing myctophids (small, mesopelagic fishes) and *M. tarapacana *containing *Sardinella *and *Cubiceps *spp. in the Philippines (Rohner et al., 2017; Stewart et al., 2017). One individual *M. tarapacana* was reported to have 27 fish in its stomach (from the Gulf of California), which were thought to be carangids (family of fish containing jacks, jack mackerels, runners, and scads), or smaller anchovy‐like species (Notarbartolo‐Di‐Sciara, 1988). Fish remains and eggs have also been observed in ray stomachs, but the particular species was not morphologically identifiable due to state of digestion, small size, or lack of identifiable characteristics (Notarbartolo‐Di‐Sciara, 1988).

DNA metabarcoding allows for high‐taxonomic resolution of diet items and are sensitive to rare species, highly degraded items, or items that leave no visual trace (Nielsen, Clare, Hayden, Brett, & Kratina, 2017). DNA metabarcoding studies have revealed insights into the dietary composition of endangered sea lions (Berry et al., 2017), exploited marine fishes (Berry et al., 2015), planktivorous fishes (Albaina, Aguirre, Abad, Santos, & Estonba, 2016) and have even been used to investigate dietary niche partitioning by large African herbivores (Kartzinel et al., 2015). These DNA‐based approaches have the potential to extend our current understanding of *Mobula* prey items and trophic interactions, especially when multiple DNA markers are combined with conventional methods (Nielsen et al., 2017; Pompanon et al., 2012). Specifically, *Mobula* rays are known to consume unspecified fish eggs and are often seen feeding around gelatinous zooplankton—jellyfish (cnidarians), comb jellies (ctenophores), and salps—which lack hard parts and can be underestimated or missed in traditional dietary analysis. Molecular techniques can reveal if these organisms are a component of ray diets. Furthermore, understanding dietary overlap between co‐occurring species can provide insight into their resource use (Foley et al., 2014), and if conducted over multiple years, can reveal if these patterns change over time (Hardy et al., 2017).

We used multiple DNA metabarcoding assays to investigate taxa in the stomach contents of four sympatric *Mobula* ray species (*M. birostris*, *M. tarapacana*, *M. japanica*, and *M. thurstoni*) caught in a direct gill net fishery off Bohol in the Philippines over a 3‐year period. These stomach contents have previously undergone morphological and stable isotope analyses, allowing for direct comparisons with these conventional methods. We identified taxa to the lowest resolution, determined the frequency of occurrence for each taxon, tested for prey differences between years, investigated dietary overlap in potential prey between species, and estimated the dietary proportions of these potential prey items.

## 2 MATERIALS AND METHODS

### 2.1 Study site and sample collection

**Figure 1:**
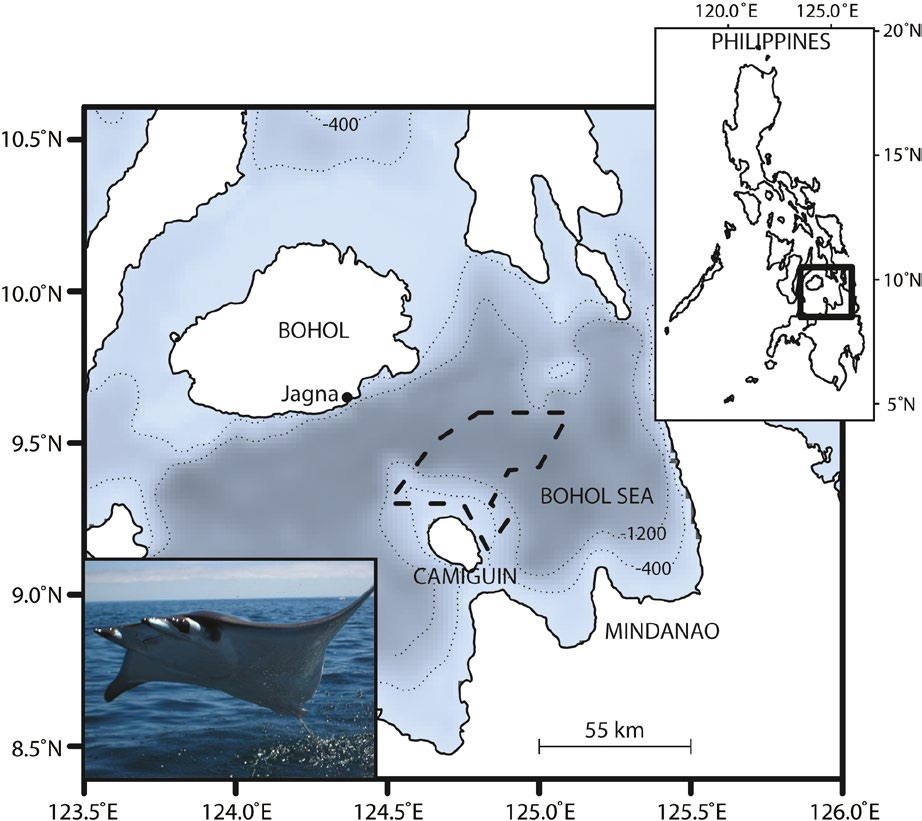
Fishing location (dashed polygon) where ray species were caught using gill nets in the Bohol Sea, Philippines. Dotted lines denote the 400 and 1,200 m isobath, with gray to blue areas denoting deepest to shallowest areas, respectively. Ray stomach content samples were extracted at the landing site in Jagna

Stomach content samples were obtained from *Mobula* rays caught by drifting gill nets in the Bohol Sea, Philippines (Figure 1), between January and June 2013–2015. Fishers stationed out of Jagna, a landing site on the island of Bohol, targeted *Mobula* rays as their main catch from well‐established fishing grounds (Figure 1, dashed lines). At the fishing grounds, fishers deployed their gill nets (~1,000–2,000 m long, 30 m high, at 10–40 m depth) which were allowed to soak at nighttime for seven hours on average. Fishers would return to the beach early the next morning to sell their catch, where the rays were measured, sexed, and their stomach content extracted (between 6 and 16 hr from capture to extraction). The whole stomach was removed (end of the esophagus to past the pyloric stomach) for conventional morphological analysis (Rohner et al., 2017), of which a homogenous 2.5 ml subsample was removed wearing gloves, preserved with ~7.5 ml of 95% EtOH, and stored in a 10 ml sterile tube for later DNA extraction and molecular analysis. Although stomach contents were typically homogeneous by nature (Rohner et al., 2017), scientists scanned the entire remains for unusual or large prey items which were not included in subsamples used for DNA extraction. Skeletal muscle tissue samples were also collected for stable isotope analysis (Stewart et al., 2017). Subsamples processed for DNA extraction were chosen to encompass an approximately even ratio of male and female specimens and span a wide range of ray sizes (Table 1).

**Table 1:** Year, sample size, timing of catch, disk width in cm (minimum, mean, maximum), and sex ratio (male:female:unknown) of all *Mobula* rays sampled

*Mobula* species	Year	*n*	Timing of catch	Disk width in cm (min, mean, max)	Sex ratio (M:F:U)
*Mobula birostris*	2013	5	January, February, April	395, 440, 524	2:2:1
	2014	12	February, April, May	380, 457, 543	4:8:0
	2015	9	February, March	231, 438, 547	4:5:0
*Mobula tarapacana*	2013	1	March	Unknown	0:0:1
	2014	8	February, March, April, May	184, 224, 271	2:2:4
	2015	15	February, March	179, 228, 279	10:4:1
*Mobula japanica*	2013	0	No catch		
	2014	7	January, February, April, May	143, 191, 234	6:1:0
	2015	18	February, March	154, 193, 232	10:5:3
*Mobula thurstoni*	2013	5	January, April, May	159, 164, 176	1:2:2
	2014	11	January, February, March, April, June	141, 160, 178	5:2:4
	2015	10	March	108, 161, 187	4:6:0

### 2.2 DNA extraction

DNA was extracted from stomach content subsamples of *M. birostris*, *M. tarapacana*, *M. japanica*, and *M. thurstoni*. Each 10 ml subsample was homogenized for one minute, and the resulting homogenate (500 μl) was collected with a wide‐bore 1,000 μl tip and pipetted into a 1.5 ml tube. Tubes were then centrifuged at 14,000 *g* (3 min), the supernatant was discarded, and the remaining sample pellet was partially dried in an Eppendorf vacuum concentrator for 3 min at 37°C. Sample pellets were then used to extract DNA according to the standard Qiagen DNeasy kit protocol for animal tissue, but with the addition of 40 µl of Proteinase K. DNA was eluted into 200 µl AE buffer (Qiagen, Venlo, the Netherlands). All extractions took place in a dedicated DNA extraction laboratory, where benches and equipment were routinely cleaned and bleached, and blank extraction controls were used.

### 2.3 Molecular analysis

A multiple metabarcoding assay approach was used to investigate the biotic diversity in ray diets. PCR was performed in duplicate on all DNA extractions using three primer sets (18S Eukaryotes, 16S Crustacea and 16S Fish) containing template‐specific oligonucleotides (Table 2; Stat et al., 2017), and fusion tag primers unique to each sample which included Illumina P5 and P7 adaptors. Performing a single round of PCR in an ultra‐clean PCR designated laboratory helped reduce the potential for chimera production, cross‐contamination, and index‐tag switching. PCR reagents included 1× AmpliTaq Gold® Buffer (Life Technologies, MA, USA), 2 μl MgCl_2 _(Applied Biosystems, MA, USA), 0.25 μl dNTPs (Astral Scientific, Australia), 1 μl of 0.4 mg/ml bovine serum albumin (Fisher Biotec, Australia), 0.4 μM forward and reverse primer, 0.6 μl SYBR® Green (Life Technologies), 0.2 μl AmpliTaq Gold DNA polymerase (Life Technologies), 2 μl of DNA, and Ultrapure™ Distilled Water (Life Technologies) made up to 25 μl total volume. Mastermix was dispensed using a Qiagility liquid handler (Qiagen), and PCR was performed on a StepOnePlus Real‐Time PCR System (Applied Biosystems) using the following conditions: initial denaturation at 95°C for 5 min, followed by 40 cycles of 30 s at 95°C, 30 s at the primer annealing temperature (Table 2), and 45 s at 72°C, with a final extension for 10 min at 72°C. All duplicate PCR products from the same subsample were combined prior to library pooling. Libraries for sequencing were made by pooling amplicons into equimolar ratios based on qPCR Ct values. Amplicons in each library were size‐selected using a Pippin Prep (Sage Science, Beverly, MA, USA) and purified using the Qiaquick PCR Purification Kit (Qiagen). The volume of purified library added to the sequencing run was determined using qPCR against DNA standards of known molarity (Murray, Coghlan, & Bunce, 2015). Depending on the amplicon size (see Table 2), libraries were either unidirectionally sequenced using a 300 cycle MiSeq® V2 Reagent Kit (for 16S Fish and Crustacea), or with paired‐end sequencing using a 500 cycle MiSeq® V2 Reagent Kit (18S Eukaryote) on an Illumina Miseq platform (Illumina, San Diego, CA, USA) located in the TrEnD Laboratory at Curtin University.

**Table 2:** Primer sets used for PCR amplification of DNA metabarcodes from ray stomach content subsamples

Primer	Oligonucleotide sequence	PCR annealing temp (°C)	Target taxa	Region	Amplicon size (bp)	Reference
18S_1F	5′ GCCAGTAGTCATATGCTTGTCT 3′	51	Eukaryotes	Nuclear	336–423	Pochon, Bott, Smith, & Wood (2013)
18S_400R	5′ GCCTGCTGCCTTCCTT 3′			18S rDNA		
16SF/D	5′ GACCCTATGGAGCTTTAGAC 3′	54	Fish	Mitochondria	178–228	Berry et al. (2017)
16S2R‐degenerate	5′ CGCTGTTATCCCTADRGTAACT 3′			16S rDNA		Deagle et al. (2007)
Crust16S_F(short)	5′ GGGACGATAAGACCCTATA 3′	51	Crustacea	Mitochondria	90–213	Berry et al. (2017)
Crust16S_R(short)	5′ ATTACGCTGTTATCCCTAAAG 3′			16S rDNA		

Blank extraction controls were included on each PCR plate, and for each different primer set. Analyses of blank controls revealed no amplification of DNA, with the exception of one sample which identified Dikarya; a Fungi (see taxonomic assignment detailed below). As a result, all Dikarya were eliminated from the analyses.

### 2.4 Data processing

Data generated by Illumina sequencing were filtered through a series of quality control steps prior to taxonomic assignment. Metabarcoding reads recovered by paired‐end sequencing were merged together using the Illumina MiSeq analysis software under the default settings. Only reads matching 100% to Illumina adaptors, index barcodes, and template‐specific oligonucleotides identified using Geneious® 8.1.4.73 were kept for downstream analyses. Reads below minimum sizes of 105, 195, and 300 bp were discarded for 16S Crustacea, 16S Fish, and 18S Eukaryote, respectively. Potential chimeras were identified using USEARCHv9.2 and removed (Edgar, 2010). Samples were collapsed into unique sequence reads and abundance filtered: a minimum of five identical reads were required to be considered for taxonomic assignment. A total of 61,227 reads (Table 3) originating from eukaryotes that passed quality filtering were queried against the NCBI (Benson et al., 2014) nucleotide database using BLASTN (Altschul, Gish, Miller, Myers, & Lipman, 1990). The search set used in BLASTN was the nucleotide collection (nr/nt), with the program selection optimized for highly similar sequences. Reads were clustered into Operational Taxonomic Units (OTUs) using the cluster_otus command (97% clustering) in USEARCHv9.2 (Edgar, 2010).

**Table 3:** Number of unique read sequences (including minimum and maximum sequence length) queried against the NCBI database for each primer set by *Mobula* species

*Mobula *species	18S Eukaryote	16S Crustacea	16S Fish
*Mobula birostris*	16,832 (308–416)	814 (151–235)	1,027 (200–224)
*Mobula tarapacana*	13,098 (312–416)	1,332 (110–172)	424 (196–224)
*Mobula japanica*	10,600 (300–417)	1,491 (160–181)	781 (198–224)
*Mobula thurstoni*	13,855 (301–427)	615 (168–172)	358 (200–224)

### 2.5 Taxonomic assignment

The taxonomic assignment of BLAST search results for each OTU was visualized using MEtaGenome ANalyser (MEGAN v. 5. 11. 3; Huson, Auch, Qi, & Schuster, 2007). Lowest common ancestor parameters were set to a max expected score of 0.01, a minimum bit score of 65, and showing the top 10% of possible matches. OTUs were resolved to genus, family, or higher, for 16S Fish or 16S Crustacea primer assays based on the percent similarity to taxa alignments; we provide a summary of maximum bit scores and identities for the most closely matched species to provide transparency in OTU clustering (Table 4). We only include taxa with ≥90% identities, and those matching online database records for fauna known to the region (e.g. Atlas of Living Australia; http://www.ala.org.au and FishBase; http://www.fishbase.org). Taxonomic assignment was restricted to order level, or higher, for the 18S universal primer assay because it is highly conserved among eukaryotes with limited power to resolve closely related taxa (Hadziavdic et al., 2014). Although all reads assigned to the host (3,097,356) were excluded (Piñol, San Andrés, Clare, Mir, & Symondson, 2014), they did act as an internal control, since each gut subsample contained a read positively identifying the known ray species. Taxonomic nomenclature was based on the World Register of Marine Species (WoRMS; http://www.marinespecies.org/).

**Table 4:** Operational Taxonomic Unit (OTU) assignments and closest database matches for reads within the OTU. Maximum bit score and identities (≥90%) for closest taxa alignments are provided

Primer	OTU assigned	Closest taxa alignments for reads within the OTU	Max bit score	Identities
18S Eukaryotes	Alveolata	Eimeriidae	590	360/377; 95%
		Oligotrichia	650	368/373; 99%
		*Colpodella tetrahymenae*	569	347/366; 95%
		Amphidinium	682	378/378; 100%
		Duboscquella	657	366/367; 99%
	Actinopterygii	*Trachurus*	688	394/400; 99%
		*Epinephelus bruneus*	616	377/399; 94%
	Doliolidae	*Doliolum*	670	383/388; 99%
	Eucestoda	*Trimacracanthus aetobatidis*	657	378/386; 98%
		*Tentacularia coryphaenae*	652	376/387; 97%
	Digenea	*Accacoelium contortum*	569	360/387; 93%
		*Gyliauchen*	565	363/396; 92%
	Acari	Histiostomatidae	661	372/376; 99%
		Cheyletidae	524	338/367; 92%
		Dermanyssina	625	363/373; 97%
	Decapoda	*Homalaspis plana*	652	382/397; 97%
	Euphausiidae	*Euphausia pacifica*	720	401/402; 100%
		*Euphausia superba*	717	400/402; 100%
		*Euphausia brevis*	708	398/402; 99%
		*Euphausia mutica*	697	389/391; 99%
		*Nematoscelis difficilis*	690	394/402; 98%
		*Nematoscelis megalops*	690	394/402; 98%
		*Nyctiphanes simplex*	684	393/402; 98%
	Brachiopoda	No close matches		
	Calanoida	*Acrocalanus monachus*	684	379/379; 100%
		*Acartia erythraea*	678	339/339; 100%
		*Nannocalanus minor*	686	380/380; 100%
	Sessilia	*Microeuraphia*	719	398/398; 100%
	Collembola	*Collembola* sp. Col_RM5	648	373/382; 98%
		*Hypogastrura *sp.	648	373/382; 98%
	Pterygota	Unclassified Elaterinae	733	406/406; 100%
		*Lepismatidae *sp.	686	388/393; 99%
	Gastropoda	Hypsogastropoda	682	380/383; 99%
		Euthyneura	693	398/405; 98%
	Stramenopiles	Bacillariophycidae	732	366/366; 100%
		Thalassiosiraceae	682	378/378; 100%
		*Chrysowaernella hieroglyphica*	641	372/383; 97%
		Oomycetes	572	357/380; 94%
	Viridiplantae	*Dunaliella*	277	161/165; 98%
		Campanulids	690	382/382; 100%
		Solanaceae	690	382/382; 100%
		*Momordica charantia*	690	382/382; 100%
16S Crustacea	Decapoda	Penaeoidea	230	155/172; 90%
	Calappidae	No close matches		
	*Euphausia*	*Euphausia recurva*	259	160/171; 94%
	*Nematoscelis*	*Nematoscelis* sp. Kcnesp	271	161/168; 96%
	*Nyctiphanes*	*Nyctiphanes australis*	302	167/167; 100%
	Unipeltata	No close matches		
	Talitridae	No close matches		
	Collembola	No close matches		
	Pterygota	No close matches		
16S Fish	Melamphaidae	*Melamphaes*	284	185/202; 92%
	*Trachurus*	*Trachurus declivis*	405	224/224; 100%
		*Trachurus japonicus*	405	224/224; 100%
	Eupercaria	*Plagiogeneion rubiginosum*	324	193/202; 96%
	*Pterocaesio*	*Pterocaesio digramma*	367	203/203; 100%
		*Pterocaesio marri*	367	203/203; 100%
	*Oxycheilinus*	*Oxycheilinus bimaculatus*	329	193/200; 97%
	*Photopectoralis*	*Photopectoralis bindus*	374	209/210; 100%
	Apogonidae	*Apogon*	262	179/199; 90%
	*Psenes*	*Psenes arafurensis*	363	201/201; 100%
	*Euthynnus*	*Euthynnus affinis*	365	202/202; 100%
		*Euthynnus lineatus*	365	202/202; 100%
	Mullidae	*Parupeneus multifasciatus*	286	185/203; 91%
	*Upeneus*	*Upeneus tragula*	365	202/202; 100%
	*Diaphus*	*Diaphus watasei*	367	203/203; 100%
		*Diaphus chrysorhynchus*	361	202/203; 99%
	Sternoptychidae	*Sternoptyx pseudodiaphana*	269	183/204; 90%
	Stomiidae	*Astronesthes chrysophekadion*	342	197/202; 98%
	Argentinidae	No close matches		
	*Sardinella*	*Sardinella lemuru*	369	204/204; 100%
		Clupeidae environmental sample	369	204/204; 100%
	*Herklotsichthys*	*Herklotsichthys quadrimaculatus*	370	208/208; 100%
	*Sardinops*	*Sardinops neopilchardus*	352	197/198; 99%
	*Encrasicholina*	*Encrasicholina heteroloba*	367	203/203; 100%

### 2.6 Proportional diet determination

Proportional diet data, for each individual, were based on the number of sequence reads assigned to each diet item divided by the total number of reads for all diet items, which enabled all stomach content subsamples to be weighted equally. Using relative sequence reads to determine diet proportions does not have a direct absolute relationship with biomass consumed (Pompanon et al., 2012), but does allow for limited estimation of relative abundance between treatments (Jarman et al., 2013) and can often provide a more accurate view of population‐level diet despite moderate recovery biases (Deagle et al., 2018).

### 2.7 Statistical analysis

Frequency of occurrence (the number of individual rays containing a prey item per ray species) was calculated for all OTUs identified in ray stomach content subsamples (Jobling et al., 2001). A Shannon–Wiener index of prey diversity was calculated for each subsample using the presence of prey OTUs identified by each of the 16S Crustacea and 16S Fish primers. As the data were not normally distributed, a Kruskal–Wallis rank sum test was then performed on the diversity index to determine significant differences between ray species or year. P‐values were adjusted with the Benjamini–Hochberg method, and a post hoc Dunn test was used to detect difference between groups. An analysis of variance was used to determine whether ray disk width was significantly related to fish prey diversity for samples with a fish prey diversity >0.

Only ray stomach content subsamples that were processed with all three primer sets (*n* = 78) were used to analyze dietary overlap and dietary proportions of potential prey, so as to ensure equivalent comparisons. Taxa identified as food items were subjected to nonmetric multidimensional scaling (nMDS) ordination using a Bray–Curtis dissimilarity matrix based on the presence/absence of taxa. A pairwise permutation MANOVA (with Bonferroni correction) was conducted in R (Vegan and RVAideMemoire packages; Dixon, 2003, Herve, 2018) to determine if ray species displayed significant differences in their diets. Similarly, these statistics were performed on proportional diet data. All graphics and statistics were produced using R (version 2.14.0; R Development Core Team 2011) and Adobe Illustrator (CC 2017).

## 3 RESULTS

### 3.1 Taxonomic assignment

**Figure 2:**
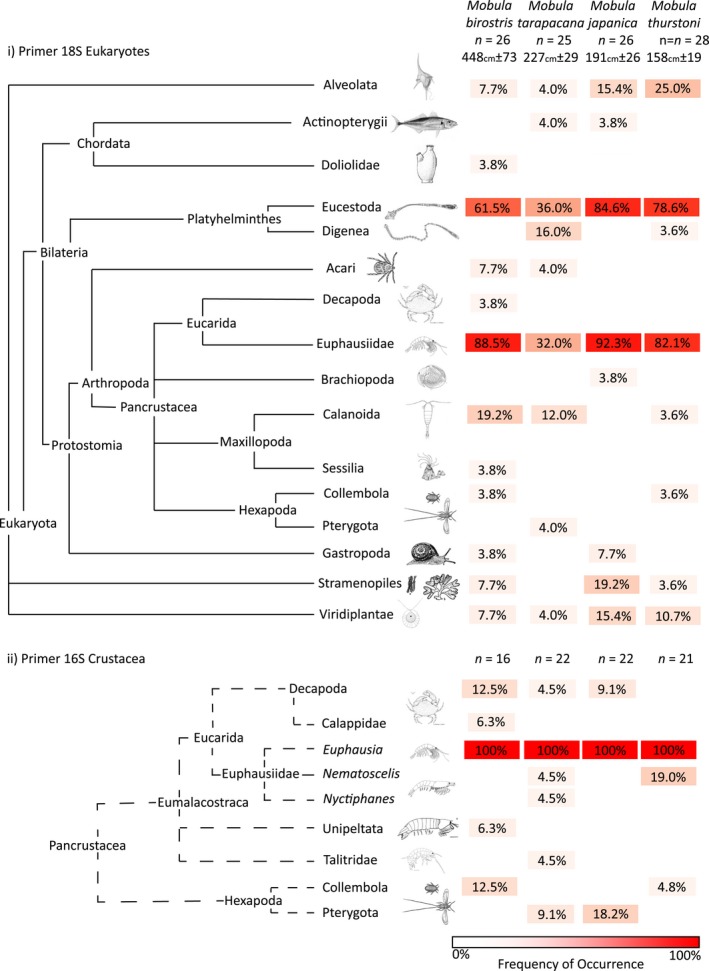
OTUs identified in all ray stomach content subsamples and frequency of occurrence for (i) 18S Eukaryote primers and (ii) 16S Crustacea primers. OTUs: Operational Taxonomic Units

**Figure 3:**
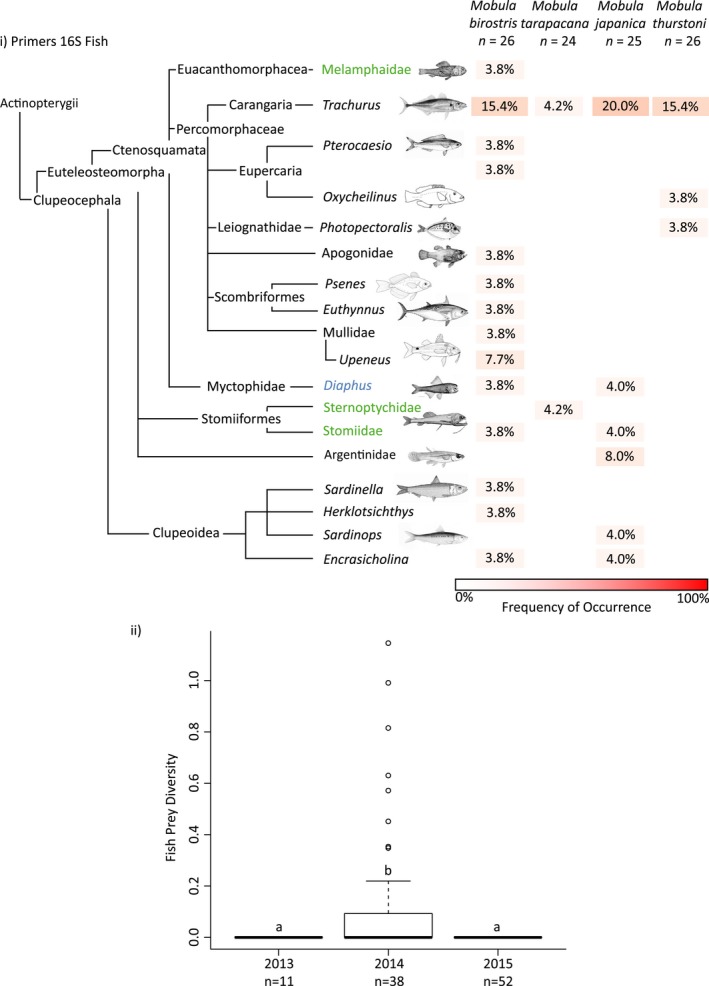
(i) OTUs identified in all ray stomach content subsamples using the 16S Fish primers and the frequency of occurrence of these assigned taxa by ray species. Sample size are provided. Deep sea, oceanic and neritic fish species are denoted in green, blue, and black, respectively. (ii) Fish prey diversity index by sampling years, where different letters indicate statistical significance at *α* = 0.05. OTUs: Operational Taxonomic Units

All subsamples yielded DNA sequences, and after sequence processing, 44 OTUs were assigned from DNA contained in the *Mobula* ray stomach contents; 16 from the 18S Eukaryote (Figure 2i), nine from the 16S Crustacea (Figure 2ii), and 19 from the 16S Fish (Figure 3i) primer assays. The closest taxa alignment for reads within each OTU is provided (Table 4). The majority of assignments for the 18S Eukaryote and 16S Fish primer assays showed ≥97% similarity to their closest taxa alignments, with some exceptions; resulting in their higher classification (see Table 4). In contrast, only four alignments showed ≥90% similarity for the 16S Crustacea primer sets, resulting in all other assignments to family or higher.

### 3.2 Frequency of occurrence of taxa and identification of possible prey items

A comparison of the frequency of occurrence of assigned OTUs for the 18S Eukaryote primer revealed four that were in common to all four ray species: Alveolata, Eucestoda, Euphausiidae, and Viridiplantae (Figure 2i). Euphausiidae had the greatest frequency of occurrence for all but one ray species. Stramenopiles (planktonic algae) and Calanoida (zooplanktonic copepods) were present in three of the ray species, with all other OTUs occurring in two or fewer ray species. The largest ray, *M. birostris*, contained 12 OTUs, with all other species containing either eight or nine OTUs. Only two ray species showed evidence of consuming bony fishes (Actinopterygii) with the 18S Eukaryote primer set.

Five OTUs were assigned to taxa that were excluded as potential prey items of rays: Eucestoda, Digenea, Alveolata, Stramenopiles, and Viridiplantae. Eucestoda (and Digenea for *M. tarapacana*) are obligate parasites which frequently occurred in the gut content of all ray species. Alveolata, Stramenopiles, and Viridiplantae could be eaten by rays, but equally could be contaminants, parasites (e.g. Eimeriidae contains common parasites of elasmobranchs), or secondarily ingested as food of the smaller filter‐feeders eaten by the rays.

Of the nine OTUs assigned for the 16S Crustacea primers (Figure 2ii), all ray species consumed *Euphausia* with a 100% frequency of occurrence. Calanoida was detected with the 18S Eukaryote primers but not with the 16S Crustacea primers. This contrasts with the other OTUs, such as Pterygota, Collembola, and Decapoda, where frequency of occurrence was greater with the 16S Crustacea primers. For example, the 16S Crustacea primers detected decapods in three ray species, whereas the 18S Eukaryote primers detected them in only one species. Crustacean prey diversity showed no significant difference between species (*χ* ^2^ = 1.447, *df* = 3, *p* = 0.695), nor years (*χ* ^2^ = 3.908, *df* = 2, *p* = 0.142).

Of the 19 OTUs assigned for the 16S Fish primers, 14 occurred in *M. birostris*, six in *M. japanica*, and three in *M. tarapacana* and *M. thurstoni* (Figure 3i). All ray species contained *Trachurus,* a carangid fish. Two of the ray species contained myctophids (genus *Diaphus*), which are oceanic (mesopelagic) fish, while three ray species contained fish from the order Stomiiformes; a group of deep‐sea fishes. All other assigned taxa of fishes were neritic in nature, with the exception of Melamphaidae (a family of deep‐sea fish). Fish prey diversity showed no significant difference between ray species (*χ* ^2^ = 5.687, *df* = 3, *p* = 0.128), but did show a significant difference between years (*χ* ^2^ = 18.153, *df* = 2, *p* < 0.001). Indeed, fish prey diversity >0 occurred only in 2014 (Figure 3b; 2013–2014, *z* = −2.556, *p* = 0.0156; 2013–2015, *z* = 0.000, *p* = 1.000; 2014–2015, *z* = 4.101, *p* < 0.001), despite the majority of sampling occurring in 2015. Of the 10 rays in 2014 displaying a fish prey diversity >0, five were mature *M. birostris* ranging from 393 to 543 cm disk width, three were immature and mature *M. japanica* ranging from 143 to 234 cm disk width, and two were *M. thurstoni* with a disk width of 162 and 164 cm. Disk width was not significantly related to fish prey diversity (ANOVA, *df* = 8, *F* = 2.001, *p* = 0.195). These 10 rays were caught in January (*n* = 1), February (*n* = 4), April (*n* = 3), and May (*n* = 2).

### 3.3 Dietary overlap and proportion of prey

**Figure 4:**
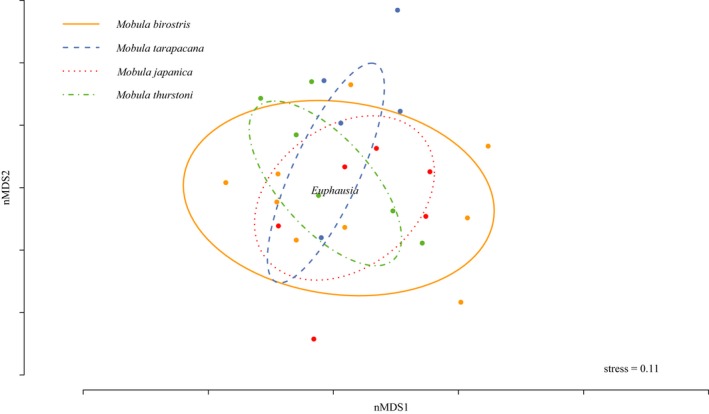
Nonmetric multidimensional scaling plot of the presence/absence of prey OTUs for each ray species. Ellipses contain 90% of the data points for each species. Only samples processed with all three primer sets are included (*n* = 78). OTUs: Operational Taxonomic Units

Ray species showed overlap in the presence/absence of prey taxa (Figure 4), with pairwise comparisons finding no significant difference between species (permutational MANOVA; all *p*‐values >0.25). *Mobula birostris* dietary space encompassed the majority of diets of all other ray species (Figure 4; nMDS where stress = 0.11, 90% of data points contained in ellipses). *Euphasia *(krill) occupied a central position in the dietary space of all ray species.

**Figure 5:**
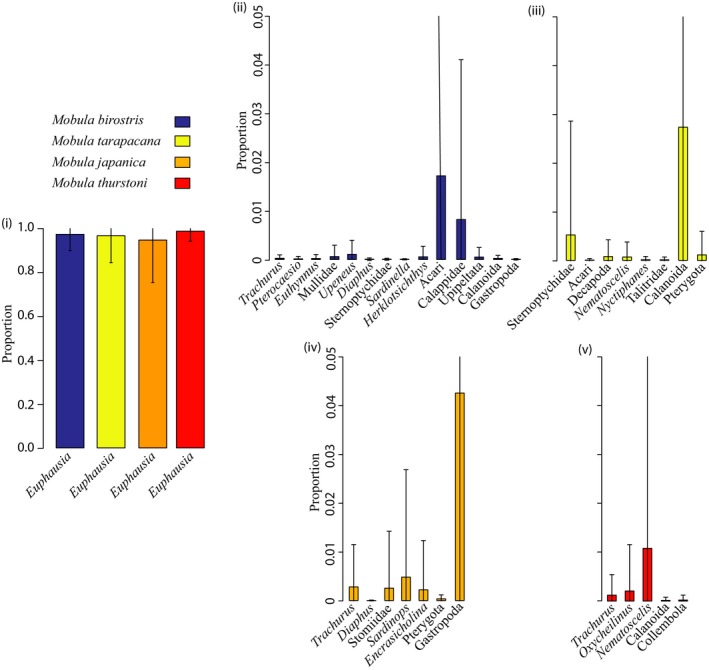
Mean proportion of DNA sequence reads corresponding to *Euphausia* (i) and all other prey taxa (*Euphausia* excluded; panes ii–v) for each ray species. Error bars are standard deviations. Only samples processed with all three primer sets are included (*n* = 78)

A total of 1,335,305 prey sequences were used in determining the proportion of diet items in ray stomach contents, of which 95% were assigned to *Euphausia*. The proportion of *Euphausia* in ray stomachs varied slightly for each ray species (Figure 5i), with no significant difference detected between species (permutational MANOVA; *p* = 0.958, *df *= 3.77; *F* = 0.795). The mean proportion of all non‐*Euphausia* diet items was <5% and highly variable (Figure 5ii–v).

## 4 DISCUSSION

The current study expands our knowledge of prey items found in *Mobula* ray diets. *Euphausia* was the main prey item by frequency of occurrence and relative sequence reads for all ray species and was detected in all samples. This finding is consistent with stomach content analyses conducted on these samples (Rohner et al., 2017), and with observations elsewhere (Notarbartolo‐Di‐Sciara, 1988). However, the diversity and incidence of bony fishes observed in ray diets are unprecedented. Also, unprecedented is the large variation in the incidence of bony fishes in the diets.

### 4.1 The presence of invertebrates in *Mobula* ray diets

Invertebrate prey identified in ray stomachs were consistent with previous studies. Indeed, conventional microscopy stomach content studies identified *Euphausia* as the dominant prey item in 91% of ray stomachs (*n* = 89, Rohner et al., 2017). We detected *Euphausia* in all stomach content subsamples when we used the 16S Crustacea metabarcoding assays. The DNA metabarcoding results were remarkably similar to the results generated by visual identification of species in stomach contents. Both methods also identified a variety of copepods and gastropods as rare diet items. The sensitivity of DNA methods allows for the detection of highly digested and unobservable species that are rarely detected by microscopy, yet no gelatinous prey items, such as jellyfish, were detected. This contrasts some recent studies of oceanic species which have identified gelatinous food items in several marine predators by DNA metabarcoding (e.g., Jarman et al., 2013, McInnes et al., 2017). It would be complimentary to take net samples in areas near feeding *Mobula *to determine whether gelatinous prey are absent in the region, or whether *Mobula *are feeding selectively and avoiding them. The detection of land‐based arthropods (Acari and Hexapoda) requires explanation since the rays were typically captured in offshore waters. Two possible explanations are as follows: first, that these organisms do not represent food items and could have come from contamination while samples were processed on the beach; and second, that they represent wind‐blown arthropods encountered when rays were feeding in nearshore surface waters. *Mobula japanica* can travel 50 km in a 24‐hr span, at speeds up to 8.3 km/hr (Freund, Dewar, & Croll, 2000), which would enable rays caught in offshore waters to have recently been feeding in nearshore areas. It has been reported that insects and land‐based arachnids are encountered as potential diet items for other nearshore, marine fish species (Berry, 1993; Hourston, Platell, Valesini, & Potter, 2004).

### 4.2 The presence of fishes in *Mobula* ray diets

Fish have previously been observed in the diets of *Mobula* rays; however, the taxonomic diversity was limited. Fish diet items were limited to myctophids, clupeids, nomeids*,* unidentified carangids, and fish larvae and eggs (Notarbartolo‐Di‐Sciara, 1988; Rohner et al., 2017; Stewart et al., 2017). We have significantly expanded upon this list, and here, we explore possible mechanisms for their presence in ray diets. One possible explanation for the majority of the fish taxa found here is that the planktonic eggs or larvae of these taxa were ingested by the rays. Eggs, although not identified as fish eggs specifically, were also found in the microscopy analysis of these samples (Rohner et al., 2017) and are often ingested by large planktivores (Robinson et al., 2013). Alternatively, fish may be directly ingested, although this is less likely given that fish bones and scales would have been observed using traditional microscopy. The genus *Trachurus*, a carangid, was detected at a 15%–20% frequency of occurrence in three ray species (*M. birostris*, *M. japanica*, and *M. thurstoni*), with the most likely species being either *Trachurus japonicus* or *Trachurus declivis*. These fish species feed on zooplankton, krill, light fish, or myctophids, on the edge of the continental shelf, where adults are commonly ~40 cm in length, and their eggs are distributed between the surface and thermocline (Maxwell, 1979). Due to the adult size of these fish, juveniles are more likely to be directly ingested by rays, rather than adults. Four species of clupeids were detected that similarly feed mainly on copepods and other zooplankton, and they are commonly found at ~20 cm in length or smaller (Whitehead, 1985), making direct ingestion by rays a possibility. Deep‐sea fishes from the order Stomiiformes were also detected in the stomachs of three ray species. Although this order of fish are benthic as adults and spawn in the deep, they migrate to near‐surface waters at night to feed on small fish and zooplankton, and their eggs likewise ascend to the near‐surface waters where they hatch (Swainston, 2011). Several taxa of more reef‐associated fish (*Eupercaria*, Mullidae, and *Euthynnus* as larvae) were also detected, all of which are known to feed on zooplankton, zoobenthos, and small fishes (Collette, 2001). DNA metabarcoding data alone does not allow for the determination of the prey’s life stage (eggs vs. larvae or juveniles), nor whether these fish were consumed selectively or incidentally while feeding upon a similar food source to the rays.

### 4.3 Temporal variation in trophic pathways

Fish prey diversity >0 occurred only in stomach content subsamples from rays caught during 2014, despite a greater number of rays being sampled in 2015. Three species of rays contained a fish prey diversity >0, and their disk width ranged from 143 to 543 cm. These data highlight the complexity and heterogeneity that can exist within trophic structure. Specific foraging location of individual rays could account for differences in fish prey diversity, whether as a result of a wider feeding area, depth range, or foraging at different times of the day. It seems unlikely that rays feeding from a common krill prey patch would encounter different diet items. Alternatively, temporal variation in fish prey availability could also explain why rays had a higher fish prey diversity in 2014. If fish eggs and larvae were more abundant in the area during 2014, this would increase their encounter rates with foraging rays. Significant reef fish connectivity throughout sites that are 100 km apart is known for this region of the Bohol Sea (Abesamis, Stockwell, Bernardo, Villanoy, & Russ, 2016). The “Bohol Jet,” a strong westward current, is hypothesized to connect multiple sites along its path (Gordon, Sprintall, & Ffield, 2011; McCook et al., 2009).

### 4.4 Dietary overlap among *Mobula* rays

There was large dietary overlap among all four species of rays. This finding is consistent with stable isotope approaches used to look at the trophic overlap among these same rays samples (Stewart et al., 2017). Stable isotope methods estimate the assimilated fraction of potential prey, and they require an understanding of the variation in isotopic values of prey items and fractionation rates; however, the relative amounts of ingested and assimilated diet can vary substantially (Bessey & Heithaus, 2015; Peterson & Fry, 1987). The stable isotope study conducted on these ray samples incorporated an understanding of the isotopic niche space and variability of several prey items, including *Sardinella*, myctophids, chaetognaths, cubiceops, euphausiids, copepods, and pteropods (Stewart et al., 2017). They observed a high degree of isotopic niche overlap between ray species, but with *M. birostris* and *M. tarapacana* having a larger isotopic niche area than both *M. japanica* and *M. thurstoni*. Although we found that *M. birostris* dietary space encompassed the majority of diets of all other ray species, we detected no significant differences between species. Fine‐scale differences in diet items can result from behavioral differences (Rohner et al., 2017; Santoro, Reiss, Loeb, & Veit, 2010; Stewart et al., 2017), small‐scale microhabitat differences in prey location, or incidental and opportunistic occurrences of alternative prey sources (Bessey & Cresswell, 2016).

### 4.5 Proportion of prey taxa in *Mobula *diets

*Euphausia* was the main prey item detected using the relative number of sequence reads. Microscopy studies on these samples found 93% of all counted prey items were *Euphausia *(Rohner et al., 2017). We likewise found 95% of all prey sequences were assigned to *Euphausia*. However, DNA sequence data cannot be used to infer absolute proportions of biomass or individuals in a pool of sequences. A number of factors bias ratios of amplicon to biomass, including primer‐binding site variation biasing the pool of sequences generated; different digestibility of prey items; and variation in DNA metabarcode density per unit biomass (Deagle et al., 2007; Thomas, Jarman, Haman, Trites, & Deagle, 2014). Nevertheless, these biases may be similar, or less, than those associated with conventional methods (Deagle & Tollit, 2007). Despite the limitations in inferring absolute biomass proportions from DNA metabarcoding data, it is still reasonable to make relative quantifications. Recent studies indicate that relative read abundance information can provide a more accurate view of population‐level diet, while studies that use frequency of occurrence alone can overestimate the importance of rare food items (Deagle et al., 2018). In our case, where we study the diet of four closely related sympatric species, biases are very likely to be consistent among the four ray species, so it is reasonable to infer that all of them eat a similar proportion of *Euphausia* and have a similarly low level of dependence on nonkrill items.

### 4.6 Caveats

Several caveats associated with our molecular approach should be acknowledged. First, gut content samples contain DNA from both consumed items, as well as from the consumer, which is usually more abundant and better preserved than those of digested prey cells (Deagle, Eveson, & Jarman, 2006). This can lead to PCR products being overwhelmed by predator sequences. Although in our study predator sequences served as a positive control, they also accounted for 70% of all sequence reads, which means that prey items may be underrepresented. Detection of prey DNA is dependent on a variety of factors, including the choice of target sequence and length, time since feeding bout, temperature, number of DNA copies, and postsampling preservation. The detection of prey DNA can be strongly attenuated directly after cessation of feeding (Weber & Lundgren, 2009), further limiting our ability to detect prey items in ray stomach subsamples since they were not preserved for up to 16 hr after ray capture. In these cases, we were likely to miss possible prey items; however, collecting gut content samples on the beach introduces an avenue for contamination, resulting in possible detections of species which are not ray prey items. Due to the sensitivity of molecular methods, it is possible to detect secondarily ingested prey items (the prey of the prey), or incidentally ingested items that are present in the water column. However, we required a minimum of five identical reads to consider the sequence for taxonomic assignment, which eliminates rare and low read sequences, thereby reducing the chances of detecting incidentally ingested items present in the water column. Taxonomic identification with our molecular approach also relies on species sequences being present within the reference database. For example, Rohner et al. (2017) were able to identify *Euphausia diomedeae* in ray stomach contents using microscopy, but the closest match we were able to obtain was a 94% identity to *Euphausia recurva*; as no reference sequence was available for *E. diomedeae* within the sequenced region.

## 5 CONCLUSIONS

This investigation has extended our knowledge on mobulid ray diet in a habitat where they are highly susceptible to exploitation. Our molecular approach recovers the diets revealed by conventional methods, but our methods also detected a greater diversity of bony fish. The increased detectability of rare bony fish prey items enabled us to identify temporal variation in trophic structure that could not be detected by morphological analyses of gut contents.

## CONFLICT OF INTEREST

None declared.

## AUTHOR CONTRIBUTIONS

OB, MB, MS, SJ, CR, AR—designed study and provided intellectual direction. CR, JR, AP—processed rays, collected samples, obtained permitting. CB, OB, AK, MP—performed molecular research. CB, OB, SJ, MS, AK, MP—processed and analyzed all data. CB—conducted statistical analysis, produced graphics, wrote manuscript, All authors contributed to manuscript revisions.

## Data Availability

Available on Dryad after manuscript acceptance.
